# Genomic features and tumor immune microenvironment alteration in NSCLC treated with neoadjuvant PD-1 blockade

**DOI:** 10.1038/s41698-021-00244-6

**Published:** 2022-01-13

**Authors:** Shuhang Wang, Pei Yuan, Beibei Mao, Ning Li, Jianming Ying, Xiuli Tao, Wei Tang, Lei Zhang, Xiao Geng, Fan Zhang, Qi Xue, Lijia Wu, Henghui Zhang, Shugeng Gao, Jie He

**Affiliations:** 1 Clinical Trial Center, National Cancer Center/National Clinical Research Center for Cancer/Cancer Hospital, Chinese Academy of Medical Sciences and Peking Union Medical College, 100021 Beijing, China; 2 Pathology Department, National Cancer Center/National Clinical Research Center for Cancer/Cancer Hospital, Chinese Academy of Medical Sciences and Peking Union Medical College, Beijing, China; 3 Genecast Biotechnology Co., Ltd, 88 Danshan Road, Xidong Chuangrong Building, Suite D 401, Xishan District, 214104 Wuxi City, Jiangsu China; 4 Nuclear Medicine Department, National Cancer Center/National Clinical Research Center for Cancer/Cancer Hospital, Chinese Academy of Medical Sciences and Peking Union Medical College, Beijing, China; 5 Radiology Department, National Cancer Center/National Clinical Research Center for Cancer/Cancer Hospital, Chinese Academy of Medical Sciences and Peking Union Medical College, Beijing, China; 6 Endoscopy Department, National Cancer Center/National Clinical Research Center for Cancer/Cancer Hospital, Chinese Academy of Medical Sciences and Peking Union Medical College, Beijing, China; 7 Thoracic Surgery Department, National Cancer Center/National Clinical Research Center for Cancer/Cancer Hospital, Chinese Academy of Medical Sciences and Peking Union Medical College, Beijing, China; 8 Biomedical Inovation Center, Beijing Shijitan Hospital, Capital Medical University, Beijing, China; 9 People’s Republic of China; School of Oncology, Capital Medical University, Beijing, China

**Keywords:** Predictive markers, Immunology

## Abstract

Several clinical trials have shown the safety and effectiveness of PD-1/PD-L1 inhibitors in neoadjuvant therapy in resectable non-small cell lung cancer (NSCLC). However, 18–83% patients can benefit from it. In this study, we aimed to assess the association of PD-L1 expression, tumor mutation burden, copy number alteration (CNA, including copy number gain and loss) burden with the pathologic response to neoadjuvant PD-1 blockade and investigate the changes in the tumor immune microenvironment (TIME) during neoadjuvant immunotherapy in NSCLC. Pre-immunotherapy treatment tumor samples from twenty-nine NSCLC patients who received neoadjuvant immunotherapy with sintilimab, an anti-PD-1 drug, were subjected to targeted DNA sequencing and PD-L1 immunochemistry staining. The pathological response was positively correlated with tumor proportion score (TPS) of PD-L1 and negatively correlated with copy number gain (CNgain) burden. Of note, the combination of CNgain burden and TPS can better stratify major pathological response (MPR) patients than did CNgain or TPS alone. Whereas, TMB showed a limited correlation with pathological regression. Additionally, PD-1 blockade led to an increase in CD8^+^PD-1^−^T cells which was clinically relevant to MPR as evaluated by multiplex immunofluorescence. A significant reduction in CD19^+^ cells was observed in the Non-MPR group but not in the MPR group, indicating the involvement of B cells in improving neoadjuvant immunotherapy response in NSCLC. Together, our study provides new data for the correlation of PD-L1 expression and genomic factors with drug response in neoadjuvant immunotherapy settings in NSCLC. The changes of TIME may provide novel insight into the immune responses to neoadjuvant anti-PD-1 therapy.

## Introduction

In the past few decades, clinical trials and studies have shown that PD-1/PD-L1 inhibitors significantly improve the survival rate of patients with advanced non-small-cell lung cancer (NSCLC)^1–4^. More recently, clinical trials of neoadjuvant immune checkpoint inhibitor (ICI) therapy have been established to provide insight into the application of this approach in resectable lung cancer. Treatment with nivolumab, atezolizumab, or sintilimab as monotherapy or in combination with other drugs was found to result in a major pathological regression (MPR) rate of 18–83%^5–11^.

Despite the encouraging results from neoadjuvant immunotherapy clinical trials, not all patients experience excellent responses, e.g., MPR or pathological complete regression (pCR). An efficient predictive biomarker would significantly improve clinical management. Although there are established immunotherapy biomarkers in metastatic disease, the association of tumor mutation burden (TMB) or PD-L1 expression with the response to neoadjuvant immunotherapy remains controversial^5–9,11^. Thus, the utility of TMB, PD-L1 expression, and other new potential biomarkers to improve the selection of patients for neoadjuvant immunotherapies needs investigation.

Immunotherapy with anti-PD-1 antibodies aims to activate a suppressed antitumor immune response in the tumor immune microenvironment (TIME). Previous studies have shown that PD-1 blockade increases the number of antigen-specific CD8^+^ T cells^12,13^, activates PD-L1^+^ natural killer (NK) cells^14^ and causes functional rebuilding of the macrophage compartment^15^. However, the impact of neoadjuvant immunotherapy on the TIME has not been fully studied.

Our previous study (ChiCTR-OIC-17013726) showed that neoadjuvant immunotherapy with sintilimab produced an MPR rate of 40.5% in NSCLC patients^6^. In the present study, 29 NSCLC patients with available pre-treatment tumor biopsy samples in this clinical trial were evaluated for genomic and immune features. Via targeted DNA sequencing of a 543-gene panel, PD-L1 IHC 22C3 pharmDx kits and multiplex immunofluorescence (mIF), we investigated the correlations of PD-L1 expression, TMB, and copy number variation (CNV) burden with the pathological response and assessed the rebuilding of the TIME by neoadjuvant PD-1 blockade.

## Results

### The association of PD-L1 expression and TMB with pathologic response to neoadjuvant immunotherapy

In total, 40 NSCLC patients were enrolled in ChiCTR-OIC-17013726. Thirty-seven patients had surgical tumor tissue samples evaluable for residual tumors^6^. Pre-sintilimab-treated primary tumors were available for 29 patients, and these samples were subjected to targeted DNA sequencing and PD-L1 IHC staining. The flow chart of the study design was shown in Supplementary Fig. 1. The clinical and pathological features of the patients were described in Supplementary Table 1. Thirty-eight percent (38%, 11/29) of the patients achieved MPR, defined as the identification of 10% or less residual viable tumor cells in the resected primary tumor. There was no significant correlation between the baseline characteristics of age, sex, smoking history or disease stage, and MPR (Supplementary Table 1).

**Fig. 1:**
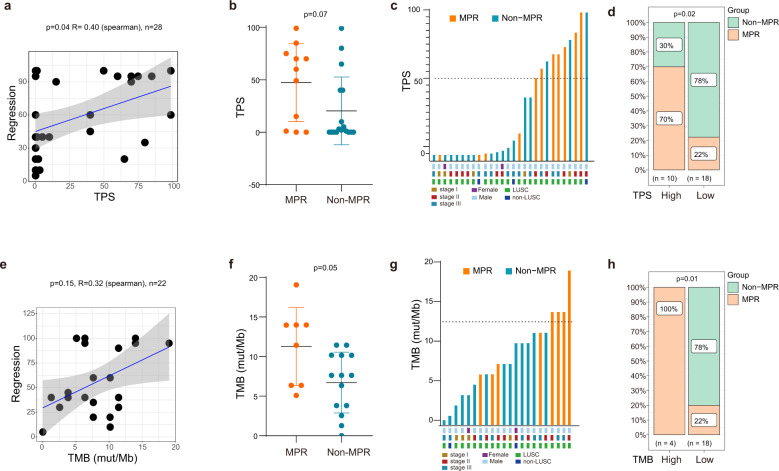
The association of PD-L1 expression and TMB with pathologic response to sintilimab. **a** The correlation between pathological regression and TPS (Spearman’s correlation coefficient R = 0.40, p = 0.04). **b** Comparison of TPS in MPR and Non-MPR group (p = 0.07, Mann–Whitney *U* test). **c**, **d** Distribution of MPR and Non-MPR patients in groups with high (≥50%) or low TPS ( < 50%) (p = 0.02, Fisher’s exact test). **e** The correlation between pathological regression and TMB (Spearman’s correlation coefficient R = 0.32, p = 0.15). **f** Comparison of TMB in MPR and Non-MPR group (p = 0.05, Mann–Whitney *U* Test). **g**, **h** Distribution of MPR and Non-MPR patients in groups with high (>12 muts/mb) or low TMB ( < 12 muts/mb) (*p* = 0.01, Fisher’s exact test).

Our previous work suggested that the expression of PD-L1 of pre-treatment tumors correlated with the degree of pathological regression in the 29 NSCLC patients when assessed by the CST anti-PD-L1 antibody (13684S)^6^. Herein, we reevaluated PD-L1 expression with the 22C3 anti-PD-L1 antibody (Dako) which is more widely used to score the expression of PD-L1 in clinical trials of immunotherapy. Due to the failure of staining for one patient, we finally obtained the TPS scores of 28 patients. TPS was positively correlated with the degree of pathologic regression (Fig. 1a, *R* = 0.40, *p* = 0.04), whereas there was no significant difference in TPS between MPR and Non-MPR patients as evaluated by Mann–Whitney *U* test (*p* = 0.07, Fig. 1b). Expression of PD-L1 scores of TPS ≥ 50%, TPS 1–49% and TPS < 1% were observed in 10, 9, 9 samples, respectively. The rates of MPR in the three groups were 70% (7/10), 22% (2/9), 22% (2/9), respectively (Fig. 1c). Thus, a TPS of 50% or higher was significantly associated with MPR by Fisher’s exact test (*p* = 0.02, Fig. 1c, d).

In addition, 22 of the 29 patients had valid targeted DNA sequencing data for the calculation of TMB, which has been approved as an immunotherapy biomarker in metastatic lung cancer^16,17^. None of the 22 patients harbored *EGFR* sensitive mutations, *ALK or ROS* fusion. There was no significant correlation between TMB and pathologic regression (*R* = 0.32, *p* = 0.15; Fig. 1e). However, marginally higher TMB was identified in patients with MPR (*p* = 0.05, Mann–Whitney *U* test; Fig. 1f). We also observed that all 4 patients with high TMB (>12 mut/Mb) achieved MPR (*p* = 0.01, Fisher’s exact test; Fig. 1g, h). However, the distribution of MPR and Non-MPR patients in the TMB high and low groups did not differ significantly when we used 11 mut/Mb or lower as the cutoff for patient stratification (data not shown), including 10mut/Mb, which has been approved by FDA for the application of pembrolizumab in solid cancers.

### CNgain burden correlates with both pathologic regression and MPR to sintilimab

**Fig. 2:**
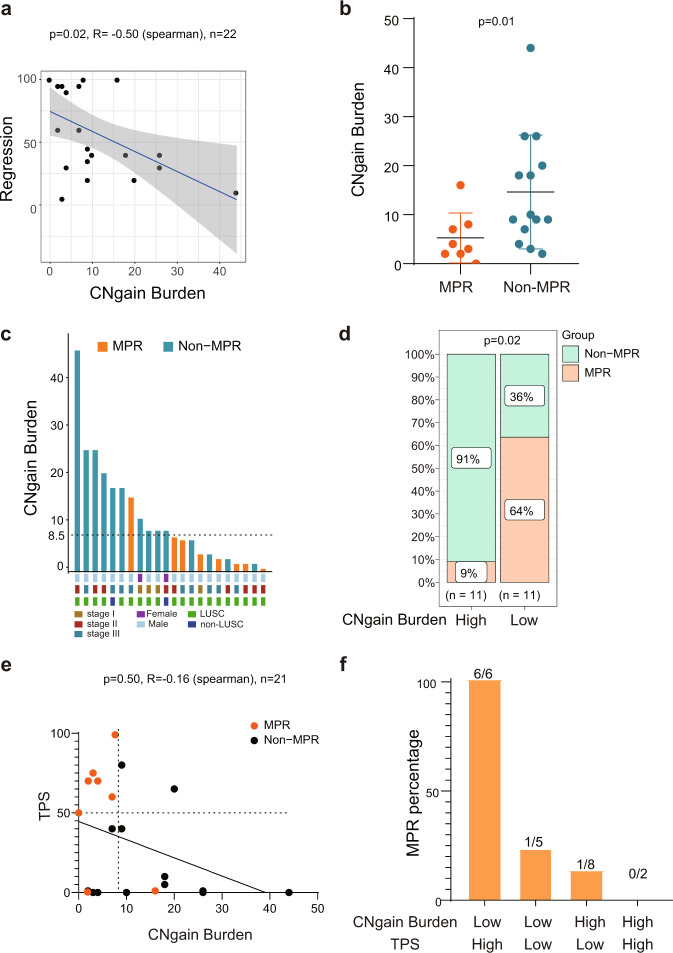
CNgain burden correlates with pathologic response to sintilimab. **a** The correlation between pathological regression and CNgain burden (Spearman’s correlation coefficient R = −0.50, p = 0.02). **b** Comparison of CNgain burden in MPR and Non-MPR group (p = 0.01, Mann–Whitney *U* test). **c**, **d** Distribution of MPR and Non-MPR patients in groups with high (≥median) or low CNgain burden (<median) (p = 0.02, Fisher’s exact test). **e** The correlation between CNgain burden and TPS (Spearman’s correlation coefficient R = −0.16, p = 0.50). **f** Rates of MPR in each of the four indicated subgroups.

The CNA burden has been reported to be associated with the clinical benefit from ICI therapy in patients with advanced NSCLC^18^. We also assessed the copy number variation and calculated the copy number alteration burden (defined as the total number of genes with copy number variation, including both gain and loss). Surprisingly, the CNA burden showed a significantly negative correlation with pathologic regression (*R* = −0.44, *p* = 0.04; Supplementary Fig. 2A). Further analysis of the number of genes with gain (CN value>3) or loss (CN value<1.5)-termed “CNgain” burden or “CNloss” burden, respectively-showed that CNgain burden (median = 8.5) had a more highly significant negative association with pathologic regression than TPS and CNA burden did (*R* = −0.50, *p* = 0.02; Fig. 2a). However, there was no correlation between CNloss burden (median = 1.5) and pathologic regression (*R* = −0.32, *p* = 0.15; Supplementary Fig. 2B). The MPR group had a significantly lower CNA burden (*p* = 0.04, Mann–Whitney *U* test; Supplementary Fig. 2C) and CNgain burden (*p* = 0.01, Mann–Whitney *U* test; Fig. 2b) than the Non-MPR group did. CNloss burden did not differ between the two groups (*p* = 0.40, Mann–Whitney *U* test; Supplementary Fig. 2D). Then, we classified the patients into a CNgain high (≥median) and a CNgain low (<median) group. Notably, 64% (7/11) of CNgain low patients were in the MPR group. Conversely, 91% (10/11) of patients with high CNgain burden did not achieve MPR (Fig. 2c, d).

Next, we attempted to obtain insight into the potential efficacy of the TPS/CNgain burden combination in discriminating high sensitivity to neoadjuvant immunotherapy. No significant correlation was observed between CNgain burden and TPS (Fig. 2e), suggesting the absence of a redundant feature between these parameters. A combined analysis revealed that the MPR rate (100%) was much higher in patients with both high TPS and low CNgain than that in patients with only high TPS or low CNgain burden (Fig. 2f).

### CNgain burden inversely correlates with the infiltration of CD8^+^ T cells in the tumor site

**Fig. 3:**
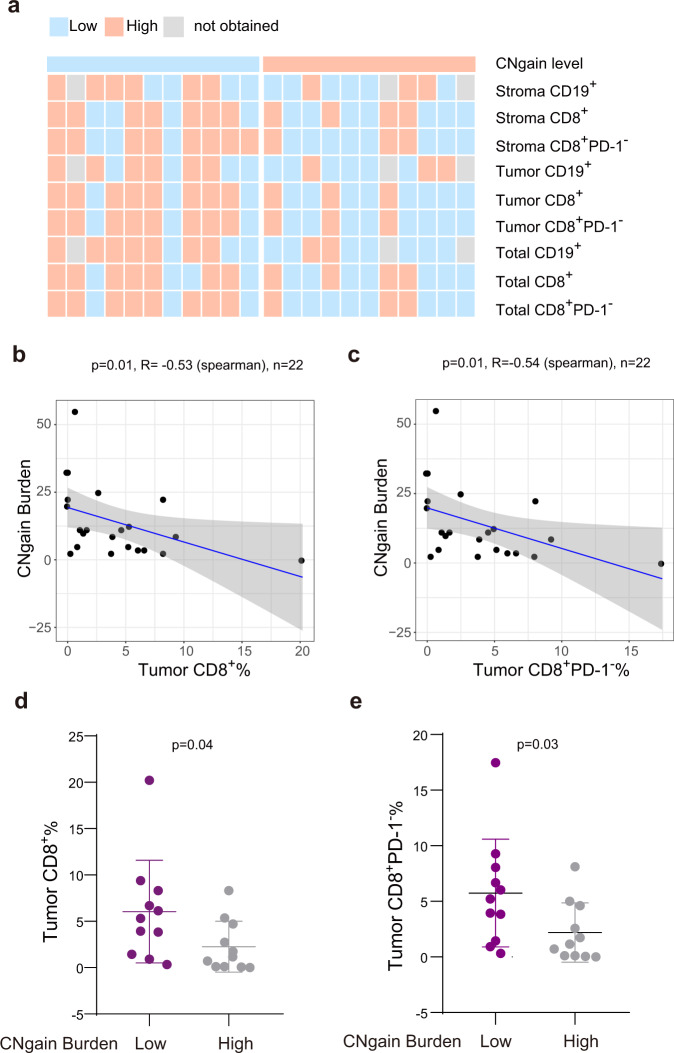
The negative correlation between CNgain burden and CD8 + cells enrichment in pre-treatment tumors. **a** Heatmap shows the distribution of indicated high and low lymphocytes in pre-treatment tumors of two groups with high or low CNgain burden. **b**, **c** The correlation between CNgain burden and CD8^+^ (**b**)/CD8^+^PD-1^−^ (**c**) T cells in tumor region of the pre-treatment samples (p < 0.05, Spearman’s correlation). **d**, **e** The comparison of CD8^+^ (**d**) / CD8^+^PD-1^−^ (**e**) T cells in CNgain low and high groups (p < 0.05, Mann–Whitney *U* test).

Recent studies have linked the copy number variation burden with antitumor immunity^19–21^. We, therefore, sought to determine the association between CNgain burden and infiltrated lymphocytes in the baseline tumors. As shown in Fig. 3a, when the patients were stratified into low and high groups according to the median abundance of the indicated lymphocyte population, most patients with low CNgain burden displayed high infiltration of CD19^+^ and CD8^+^ T cells. In particular, CNgain burden had a significantly inverse correlation with infiltration of CD8^+^ and CD8^+^PD-1^−^ T cells in tumor region (Fig. 3b, c). In addition, the proportions of CD8^+^ and CD8^+^PD-1^−^ T cells in the tumor region were significantly higher in the CNgain burden low group than those in the CNgain burden high group (*p* < 0.05, Fig. 3d, e). Collectively, the histological and mIF staining data confirmed the negative correlation of copy number variation with an activated inflammatory response in the TIME, which was previously discovered based on RNA-seq data^21^.

Taken together, the above data indicated the associations of PD-L1 expression, TMB and copy number variation, with the response to neoadjuvant immunotherapy in NSCLC patients.

### Rebuilding of the TIME by neoadjuvant anti-PD-1 therapy

**Fig. 4:**
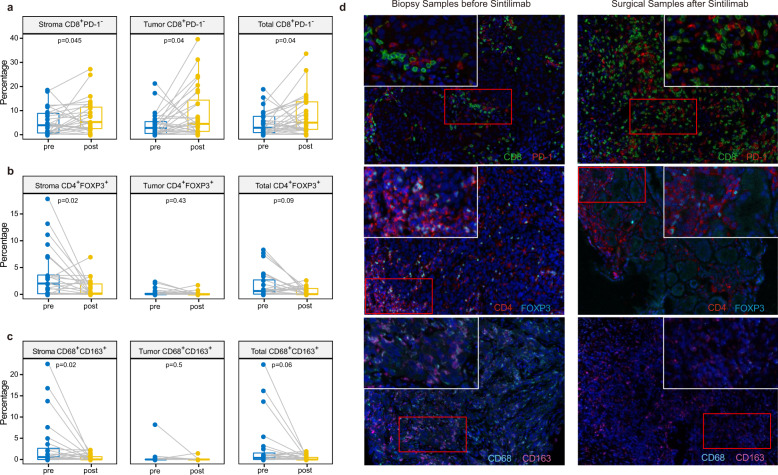
Neoadjuvant anti-PD-1 blockade alters the immune cell infiltration in the tumor microenvironment. **a**–**c** Changes in the composition of indicated immune cells infiltration in stromal, tumor and total regions from pre- to post-immunotherapy samples (Mann–Whitney *U* test for paired samples). **d** Representative mIF staining figures of a patient with MPR depict the changes in presence of various immune cell types in response to sintilimab blockade. In the boxplots in this figure, the center line represents the median value, the bounds of the box represent the interquartile range, and the whiskers extend to 1.5× the interquartile range on either side of the median.

By analyzing both pre-treatment and post-treatment (surgical) tumor samples, we aimed to examine the impact of neoadjuvant anti-PD-1 therapy on the TIME in 29 patients. The proportion of overall CD8^+^ T cells tended to be increased by PD-1 blockade therapy (Supplementary Fig. 3A–C). Among CD8^+^ cells, the proportion of CD8^+^PD-1^−^ cells (Fig. 4a) were increased in post-treatment samples. In contrast, the proportions of CD4^+^ T and CD68^+^ cells were reduced in the stromal region of post-treatment samples (*p* = 0.04 for CD4^+^ T cells, *p* < 0.01 for CD68^+^ cells, Mann–Whitney *U* test for paired samples; Supplementary Fig. 3A). The results of co-staining of FOXP3 with CD4 or CD163 with CD68 showed that the proportions of two subtypes of immune suppressor cells, Tregs (CD4^+^FOXP3^+^) and M2 macrophages (CD68^+^CD163^+^), were reduced in the stromal region of post-treatment samples (*p* < 0.05, Mann–Whitney *U* test for paired samples; Fig. 4b, c). However, after anti-PD-1 treatment, the proportion of B cells (CD19^+^) was marginally reduced in the stromal region (*p* = 0.06, Mann–Whitney *U* test for paired samples; Supplementary Fig. 3A). Representative mIF staining images from patients with MPR are displayed in Fig. 4d.

### The sintilimab-induced increase of CD8^+^ T cell and reduction of CD19^+^ cell proportion in the TIME is associated with pathologic response

**Fig. 5:**
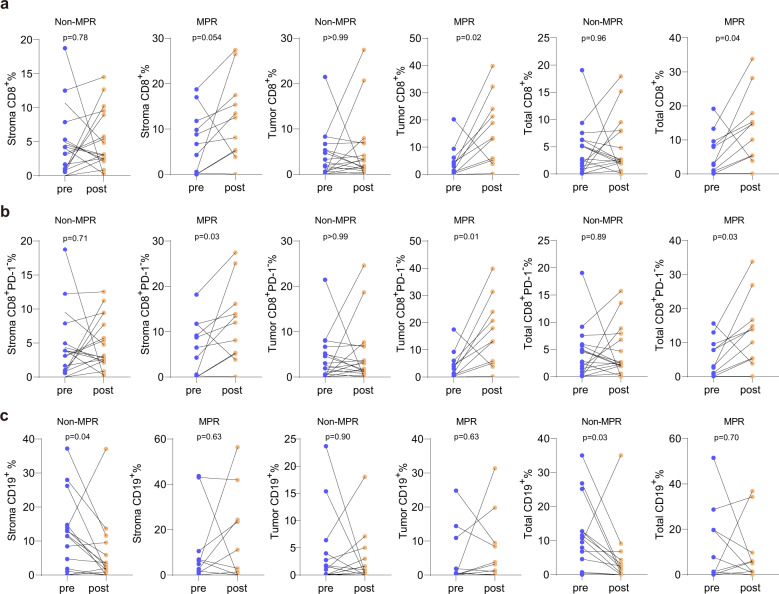
The change of CD8^+^ and CD19^+^ cells was distinct in MPR and Non-MPR patients. **a** Analysis of changes in CD8^+^ T cells infiltration in MPR and Non-MPR patients (Mann–Whitney *U* test for paired samples). **b** Analysis of changes in CD8^+^PD-1^−^ T cells infiltration in MPR and Non-MPR patients (Mann–Whitney *U* test for paired samples). **c** Analysis of changes in CD19^+^ cells infiltration in MPR and Non-MPR patients (Mann–Whitney *U* test for paired samples).

Subsequently, we sought to determine whether the changes in the enrichment of cytotoxic CD8^+^ T cells, Treg cells, and macrophages were correlated with pathological response to neoadjuvant PD-1 blockade. As the correlation analysis results are shown in Supplementary Fig. 4A, we found no significant association between the degree of pathological regression and changes in overall lymphocyte infiltration (*p* > 0.05, Spearman correlation, Supplementary Fig. 4A). However, the degree of increase in the proportion of CD8^+^PD-1^−^ T cells in MPR patients was significantly higher than that in Non-MPR patients (*p* < 0.05, Mann–Whitney *U* test; Supplementary Fig. 4B). Notably, anti-PD-1 therapy elicited enrichment of CD8^+^ and CD8^+^PD-1^−^ T cells only in MPR patients (paired Mann–Whitney *U* test, Fig. 5a, b), indicating a critical role of non-exhausted CD8^+^ T cells in the effectiveness of neoadjuvant PD-1 blockade. In addition to CD8^+^ T cells, we also found that a significant reduction in CD19^+^ cells in the stromal and total regions occurred only in Non-MPR patients, not in MPR patients (Fig. 5c), suggesting the contribution of B cells to the MPR resulting from neoadjuvant immunotherapy.

## Discussion

In this study, we characterized the associations of PD-L1 expression and genomic features with the response to neoadjuvant ICI therapy and illustrated the dynamic change in immune cell infiltration during neoadjuvant immunotherapy. PD-L1 expression in baseline tumors has been evaluated in several NSCLC neoadjuvant immunotherapy trials, including NEOSTAR^7^, NADIM^8^, and LCMC3^9^. Similar to our findings, all three reports indicated that a high level of PD-L1 expression was associated with the response to neoadjuvant immunotherapy. However, the association of TMB with the response in the neoadjuvant ICI setting remains controversial in NSCLC. The LCMC3 trial, which evaluated atezolizumab monotherapy, reported that TMB was not different between the MPR and Non-MPR groups^9^. Whereas, among patients treated with nivolumab monotherapy, a significantly higher mutation burden was observed in tumors from patients with MPR^5^. Our data that in early-stage NSCLC, MPR was slightly associated with high TMB suggests the limitation of TMB in identifying MPR patients.

More importantly, we assessed CNA burden in neoadjuvant ICI therapy for the first time. Our DNA sequencing data demonstrated strong inverse correlations between CNA burden and pathologic regression or MPR. Specifically, CNgain burden but not CNloss burden contributed to the correlation between CNA and the efficacy of neoadjuvant ICI therapy. In metastatic NSCLC, two studies have reported that the somatic CNA (SCNA) burden was lower in responders than that in non-responders^18,22^. While, a study in melanoma found a higher burden of copy number loss but not copy number gain in non-responders to CTLA-4 and PD-1 blockade^20^. This finding combined with our results indicated the distinct roles of CNgain and CNloss burdens in mediating the response to ICI therapy in different cancers.

Compared with tumors with low aneuploidy, tumors with high aneuploidy exhibit uncontrolled cell proliferation^21^. In addition, integrative analyses based on TCGA data sets have demonstrated a negative correlation between CNA burden and cytotoxic immune infiltrates^21,23,24^. Consistent with previous findings, the baseline tumor samples with low CNgain burden in our study exhibited higher infiltration of CD8^+^ cells in the tumor region than those with high CNgain burden did, as visualized by mIF. This further clarifies the association between CNA burden and antitumor immunity. Therefore, in our analysis combining TPS with CNgain burden, 100% of TPS^high^CNgain^low^ patients were found to achieve MPR. High expression of PD-L1 indicates that immune escape contributes to the progression of these tumors. On the other hand, a concurrent low CNA burden indicates a relatively low proliferation rate and an increased proportion of CD8^+^ cells, thus permitting a rapid and strong response to ICI therapy. Despite these discoveries, the associations of TMB, PD-L1 expression and CNgain burden with neoadjuvant immunotherapy need to be validated in large cohorts with more NSCLC patients, e.g., phase II or III clinical trials. The prospective multicenter trials for neoadjuvant immunotherapy with the include and exclude criteria based on the status of these biomarkers are still necessary for the clinical translational study.

We also examined the possibility that neoadjuvant PD-1 blockade can rebuild TIME to enhance anticancer immune response^25,26^. Several studies revealed that CD8^+^ T cells accumulated in peripheral blood and tumor site in response to anti-PD-1 therapy in advanced cancers^27,28^. The increased CD8^+^ cells could probably derive from the expansion of CD8^+^ cells which lack expression of PD-1 and other checkpoint receptors^13^. Herein, in response to neoadjuvant anti-PD-1 blockade, we observed an obvious elevation of CD8^+^PD-1^−^ cells. This alteration of CD8^+^PD-1^−^ cells was associated with a major pathological response, clarifying the importance of non-exhausted CD8 cells in neoadjuvant anti-PD-1 blockade of early-stage NSCLC. In contrast to CD8^+^ cells, Tregs and M2 macrophages were reduced in response to neoadjuvant immunotherapy administration. Tregs inhibit the antitumor function of immune effector cells and take part in tumor immune escape^29,30^. M2 macrophages also exert the immunosuppressive function through anti-inflammatory cytokine secretion and wound healing regulation^31^. The suppression of Tregs and M2 macrophages ameliorates the TIME. In addition to T cells, B cells are main components of the adaptive immune system. Two studies in melanoma discovered the role of B cells in response to ICI therapy^32,33^. Our research found that CD19^+^ B cells were reduced only in Non-MPR patients, implying the function of B cells in the maintenance of antitumor activity in NSCLC patients. Taking together, neoadjuvant anti-PD-1 blockade improves the inflamed immune microenvironment of patients, thereby accelerating the T cell activation and tumor killing.

Although the findings from this study are exciting, we would also like to point out several limitations. First, the small sample size limits the power of the conclusions, especially when multiple factors and subgroup analyses are considered. Second, we need to continue follow-up and assess the prognostic value of PD-L1 expression, TMB and CNA burden in neoadjuvant ICI therapy. The retrospective nature is another limitation; tissue samples were not available for all patients treated in this clinical trial. Moreover, other methods, e.g., single-cell sequencing assay and spatial transcriptomics would be helpful for the precise elucidation of TIME rebuilding in response to neoadjuvant immunotherapy in NSCLC.

Our study provides data about PD-L1 and CNgain burden as potential selective indicators for single PD-1 inhibitor neoadjuvant treatment in NSCLC patients. In parallel, the examination of dynamic changes in the TIME during treatment suggests neoadjuvant immunotherapy impacts on multiple cell types to activate antitumor immunity.

## Methods

### Study design and patient samples

In all, 40 NSCLC patients were enrolled in the clinical trial ChiCTR-OIC-17013726 (a prospective single-centre, single-arm phase Ib study)^6^. Among them, 29 patients with available pre- and post-treatment samples were included for the analysis of genomic and immunologic features. All patients had measurable disease at baseline. The RECIST 1.1 guidelines were used to assess the radiographic response to neoadjuvant immunotherapy. The percentage of residual viable tumor at the primary site was identified by routine hematoxylin and eosin staining. Tumors with no more than 10% viable tumor cells were considered to exhibit major pathological responses. The methods used to assess pathological response has been published previously^34^.

The clinical trial was conducted in accordance with the Declaration of Helsinki and the International Conference on Harmonization Guidelines for Good Clinical Practice. The Ethics Committee and Institutional Review Board of National Cancer Center/Cancer Hospital, Chinese Academy of Medical Sciences and Peking Union Medical College approved this prospective study and written informed consent was obtained from patients.

### Targeted DNA sequencing

DNA extraction, library preparation, sequencing, and somatic mutation calling were conducted as described previously^35^. In total, 543 genes associated with cancer diagnosis and prognosis were identified.

After filtering germline mutations, SNV mutations were selected from all samples according to the following rules: (i) mutations affecting splicing or located in an exonic region; (ii) mutations with a depth of ≥100× and an allele frequency of ≥5%; (iii) mutations with an allele frequency of ≥0.2% in the Exome Aggregation Consortium (ExAC) and the Genome Aggregation Database (gnomAD); and (iv) mutations without strand bias. Paired blood gDNA samples were used as controls to distinguish somatic mutations from inherited germline variations.

Subsequently, the absolute mutation counts of the tumor samples were used to calculate TMB with the following formula: Absolute mutation count × 1000000/Panel exonic base num. TMB was measured in mutations per Mb.

We used CNVkit (v0.9.2) to obtain the copy number value from the tumor samples for each patient and each gene. A panel of blood samples from healthy control individuals was used for reference construction. A gene was considered to have a copy number gain >3 or loss <1.5 only if the number of target intervals was ≥5.

CNA burden was defined as the total number of genes with copy number alteration per sample. CNgain/loss burden was defined as the total number of genes with copy number gain or loss per sample.

### PD-L1 staining

Analysis of PD-L1 expression was conducted according to instructions of the Dako 22C3 kit. The tumor proportion score (TPS), defined as the percentage of all living tumor cells with partial or complete staining of the membrane, was used to evaluate PD-L1 expression.

### Multiplex immunofluorescence staining assay

The staining was performed manually. For multiplex fluorescence immunohistochemical staining, paraffin-embedded NSCLC tissue blocks were serially sectioned into 3 µm sections. The slides were deparaffinized, rehydrated, and subjected to epitope retrieval by boiling in Tris-EDTA buffer (pH = 9; Klinipath #643901, Duiven, the Netherlands) for 20 min at 97 °C. Endogenous peroxidase was then blocked by incubation in Antibody Diluent/Block (PerkinElmer #72424205, Massachusetts, USA) for 10 min. Only one antigen was detected in each round, including primary antibody incubation, secondary antibody incubation, tyramine signal amplification (TSA) visualization, followed by labeling of the next antibody after epitope retrieval and protein blocking as before. Primary antibodies for FOXP3 (1:400; ab20034; Abcam), CD4 (1:50; ZM0418; Zsbio, China), CD68 (1:500; ZM0060; Zsbio, China), CD163 (1:100; ZM0428, Zsbio, China), CD19 (1:100; ZM0038, Zsbio, China), CD8 antibody (1:50; ZA-0508, clone SP16, Zsbio), and PD-1 (1:100; ZM0381, Zsbio, China) were incubated for 1 h at room temperature. CD56 (1:50; ZM0057, Zsbio, China) were incubated overnight at 4 °C. Samples were then incubated at 37 °C for 10 min with the antibody using Opal ploymer anti-rabbit/mouse horseradish peroxidase (HRP) Kit (PerkinElmer #2414515, Massachusetts, USA). TSA visualization was then performed with the opal seven-color multiplex immunohistochemistry Kit (NEL797B001KT, PerkinElmer, Massachusetts, USA), containing fluorophores (DAPI), Opal 690 (CD163), Opal540 (CD56, CD8), Opal 620 (CD19, CD4), Opal 650 (CD68, FOXP3, PD-1), and TSA Coumarin system (NEL703001KT, PerkinElmer, Massachusetts, USA). After labeling for all the antigens for each panel, microwave treatment (MWT) was performed to remove the TSA-antibody complex with Tris-EDTA buffer (pH = 9; Klinipath #643901, Duiven, the Netherlands) for 20 min at 97 °C. All the slides were counterstained with 4′,6-Diamidino-2-Phenylindole (DAPI) for 5 min and were enclosed in Antifade Mounting Medium (NobleRyder #I0052, Beijing, China), prepared for imaging. Quality control samples of tonsil tissue from the autopsy were included in each staining batch as positive control and to assess the interexperimental reproducibility.

Due to sample falling off slide caused by insufficient adhesion, we only obtained paired pre-post tumor staining data of panel (CD56, CD19, CD68, CD163) for 24 patients, panel (CD8, PD-1) for 28 patients and panel (CD4, FOXP3) for 25 patients.

### Tissue imaging and analysis

Slides were scanned using a PerkinElmer Vectra imaging system (Vectra 3.0.5; PerkinElmer, Massachusetts, USA). After a low magnification scan (4x or 10x), regions of interest (ROI) were selected using the Phenochart viewer (Akoya Bioscience) and these ROIs were subsequently scanned/acquired at a higher resolution (20x) and subjected to inForm analysis. Multispectral images were unmixed using spectral libraries constructed from images of tissues stained separately with each reagent using inForm Advanced Image Analysis software (inForm 2.3.0; PerkinElmer, Massachusetts, USA). A selection of 5–10 representative original multispectral images was used to train the inForm software (tissue segmentation, cell segmentation, phenotyping tool, and positivity score). All settings applied to the training images were saved in an algorithm to allow the batch analysis of multiple original multispectral images of the same tissue.

The percentage of positively stained cells was calculated as the number of positively stained cells/The total number of cells with a nucleus.

### Statistical analysis

Categorical variables were compared between two groups using Fisher’s exact test or the chi-square test. For continuous variables, differences in the means or medians between two groups were assessed by the Mann–Whitney *U* test. Correlation coefficients were computed by Spearman correlation analysis. All statistical analyses were carried out using GraphPad Prism 8 or R 3.6.1. All significance tests were two-tailed, and differences with *p* < 0.05 were considered statistically significant.

### Reporting summary

Further information on research design is available in the Nature Research Reporting Summary linked to this article.

## Supplementary information

Supplementary materials

REPORTING SUMMARY

## Data Availability

The targeted DNA sequencing data has been submitted to GSA-Human Database (https://ngdc.cncb.ac.cn/gsa-human). The accession number is HRA000900.
