# Supplementary material for: Genomic features and tumor immune microenvironment alteration in NSCLC treated with neoadjuvant PD-1 blockade
Source: NPJ Precis Oncol. 2022 Jan 13;6:2. doi: 10.1038/s41698-021-00244-6 (PMC8758728; doi:10.1038/s41698-021-00244-6)
Supplement: Supplementary file 1 — Supplementary materials [file 41698_2021_244_MOESM1_ESM.docx]

**Supplementary Table 1. Association Between Clinical Characteristics and MPR.**

| Characteristics | MPR (%) | Non-MPR (%) | p Value |
| --- | --- | --- | --- |
| Gender |  |  |  |
| Male | 11 (100) | 15 (83) | 0.27^a^ |
| Female | 0 (0) | 3 (17) |  |
| Age |  |  |  |
| Median | 57 | 62.5 | 0.26^b^ |
| Histology |  |  |  |
| Squamous | 11(100) | 14(78) | 0.27^a^ |
| Nonsquamous | 0(0) | 4(22) |  |
| Stage |  |  |  |
| I | 1 (10) | 4 (22) | 0.78^a^ |
| II | 5 (45) | 8(44) |  |
| III | 5(45) | 6(33) |  |
| Smoking |  |  |  |
| Smoker | 11(100) | 15(83) | 0.27^a^ |
| Non-smoker | 0 (0) | 3(17) |  |
| Radiographic Response |  |  |  |
| PR | 4(36) | 1(6) | 0.085^a^ |
| SD | 7 (64) | 14(78) |  |
| PD | 0 (0) | 3(17) |  |

^a^p value is calculated using Fisher’s exact test.

^b^p value is calculated using Mann Whitney *U* test.

Abbreviations: PR: partial response; SD: stable disease; PD: progressive disease.

**Supplementary Figures**

**
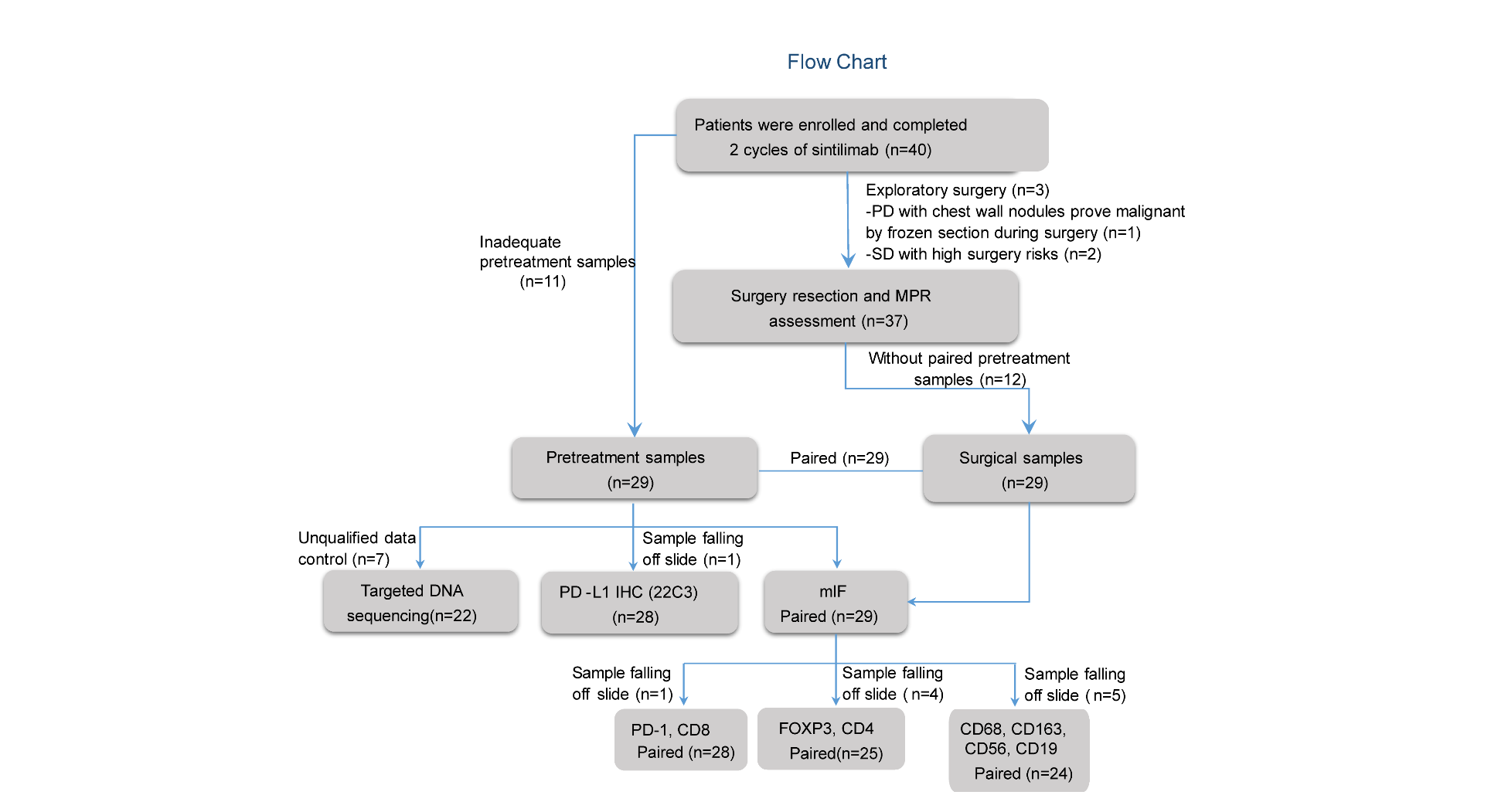
**

**Supplementary Figure 1. Description of the schematic of sampling strategy and experimental workflow.**

**
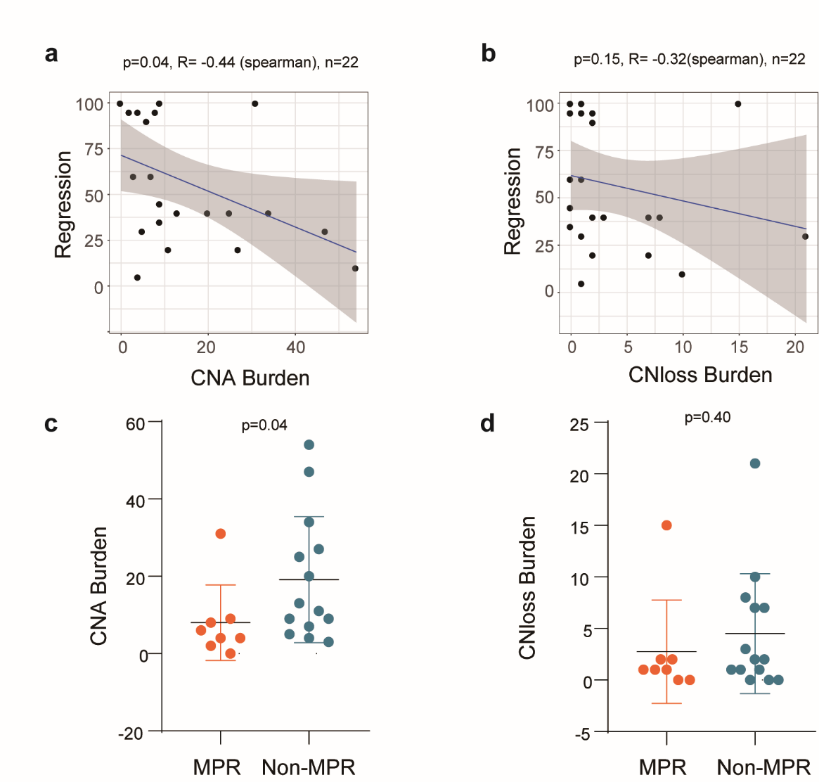
**

**Supplementary Figure 2. The association of CNA burden and CNloss burden with pathologic response to sintilimab.**

(a-b) The correlation between CNA burden (a) / CNloss burden (b) and pathological regression (Spearman's correlation). (c-d) Comparison of CNA burden (c) and CNloss burden (d) in MPR and Non-MPR group (Mann Whitney *U* test).


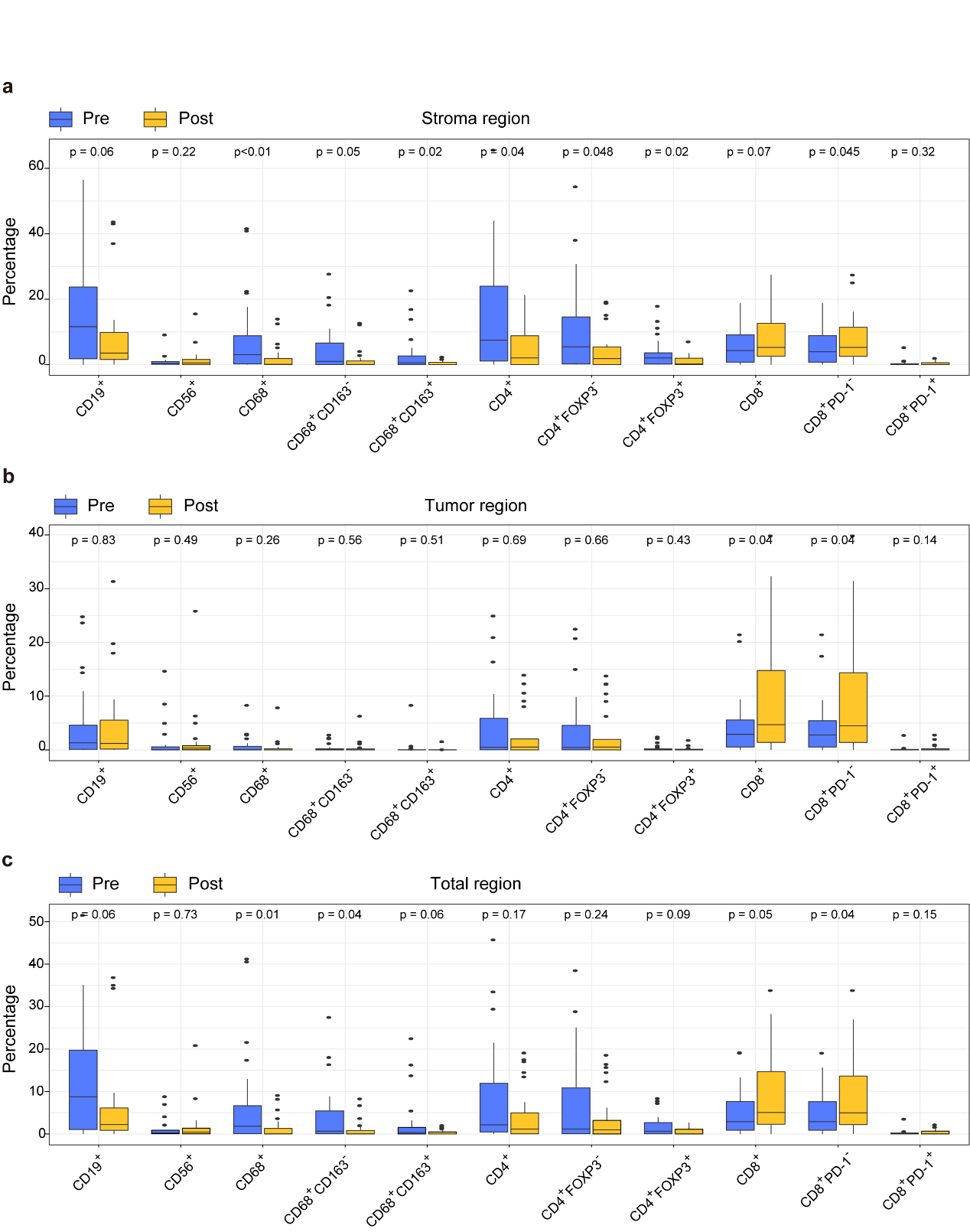


**Supplementary Figure 3. The alteration of immune cells infiltration induced by neoadjuvant administration of sintilimab**.

Comparison of indicated immune cells abundance in stroma(a), tumor (b) and total region (c) of the pre- and post-immunotherapy samples. In the boxplots in this figure, the center line represents the median value, the bounds of the box represent the interquartile range, and the whiskers extend to 1.5× the interquartile range on either side of the median.


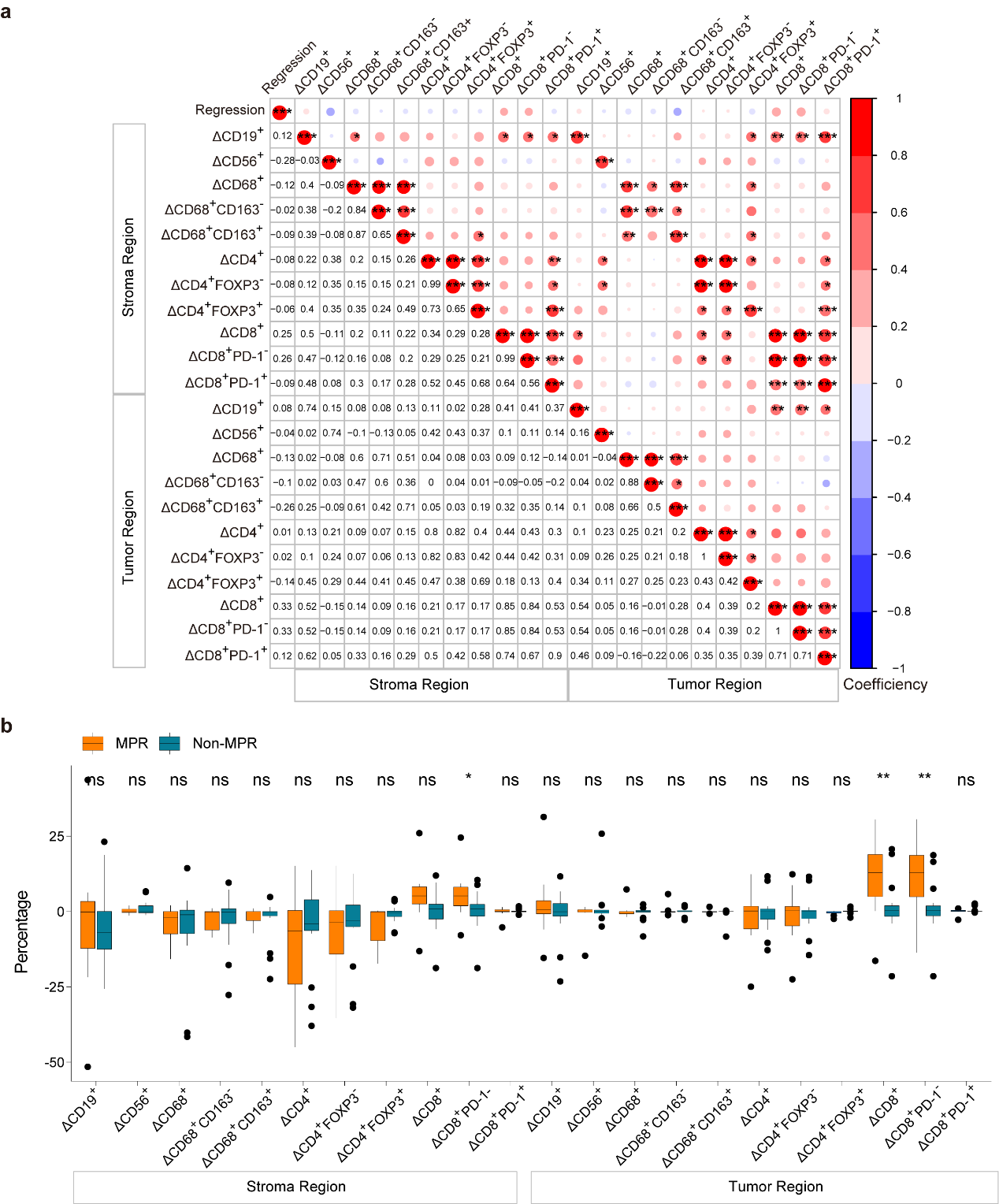


**Supplementary Figure 4. The association of changes in the composition of the immune cell infiltrate with pathologic response.**

(a) Bubble plot showing the correlation between pathological regression and changes of indicated immune cell types (*, p<0.05; **, p<0.01; ***, p<0.001; Spearman’s correlation). (b) Comparison of the composition alteration of the immune cell infiltrate between MPR and Non-MPR groups in response to neoadjuvant immunotherapy (*, p<0.05; **, p<0.01; ns, not significant; Mann Whitney *U* test). In the boxplots in this figure, the center line represents the median value, the bounds of the box represent the interquartile range, and the whiskers extend to 1.5× the interquartile range on either side of the median.
